# A Report of Four Cases of Blackwater Fever after Quinine Treatment at Zinder National Hospital, Niger Republic

**DOI:** 10.1155/2019/2346087

**Published:** 2019-08-25

**Authors:** Doutchi Mahamadou, Diongolé M. Hassane, Moussa Tondi Maiga Zeinabou, Iliassou Aboubacar, Ali Osseini, Adamou Harissou, Garba Abdoul-Aziz, Alkassoum Ibrahim, Ibrahim Maman Laminou, Adéhossi Eric

**Affiliations:** ^1^National Hospital of Zinder, Zinder, Niger; ^2^Faculty of Health Sciences, University of Zinder, Zinder, Niger; ^3^Faculty of Health Sciences, University of Niamey, Niamey, Niger; ^4^Centre de Recherche Médicale et Sanitaire, Niamey, Niger

## Abstract

**Background:**

Blackwater fever (BWF) is a rare but serious complication of malaria that is a consequence of antimalarial treatment. Its prevalence seems to have increased. Its diagnosis is based on clinical symptoms and urine color. We report on 4 BWF cases admitted to the infectious diseases department of Zinder National Hospital.

**Results:**

Four patients were hospitalized in September 2017 for a hepatorenal syndrome of jaundice, port wine-colored urine, renal failure, and hepatic cytolysis following antimalarial treatment with quinine salts. Quinine treatment was stopped and treatment was continued with injectable artemether. Three patients underwent extra-renal purification. Their evolution was favorable. One patient died less than 24 hours after admission.

**Conclusion:**

A rare and severe complication, blackwater fever must be considered for patients under antimalarial treatment who present with jaundice, abdominal pain, and acute renal insufficiency with port wine-colored urine. Rapid diagnosis and management in an intensive care unit are crucial for improving the prognosis.

## 1. Introduction

Blackwater fever (BWF) is an acute intravascular hemolysis that results from antimalarial drug use [1–3]. It clinically manifests as hemolytic anaemia, hemoglobinuria, acute renal failure, and hypovolemia [3]. Well known in the early twentieth century, blackwater fever became rare after 1950 with the replacement of quinine by chloroquine. This disease reappeared around 1990, following the reintroduction of quinine due to chloroquine resistance. Several cases have been described with halofantrine and mefloquine, two new molecules close to quinine (family of amino alcohols) [4–6]. The treatment with lumefantrine was also associated with BWF [7, 8]. Some have linked this condition to viral and bacterial infections [9, 10].

Glucose-6-phosphate dehydrogenase deficiency (G6PD) also is frequently associated with this syndrome; however, its role is not clearly demonstrated [1, 6]. More recently, cases have been reported in patients taking artemisinin for malaria treatment [1, 7, 8]. Also, certain viral and bacterial diseases have been linked to the occurrence of BWF [9, 10]. Its pathophysiology thus remains unclear [1, 9, 10]. In the present study, we report on four blackwater fever cases in adults treated with quinine for severe malaria.

## 2. Observations

### 2.1. First Observation

**Figure 1:**
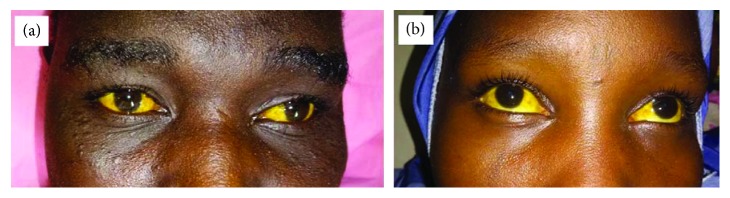
Conjunctival jaundice found in patients.

A 40-year-old male patient was referred to Mirriah District Hospital located at 20 km from Zinder, the regional capital, for a fever associated with vomiting and sudden onset of a consciousness disorder. A week earlier, he had received malaria rapid diagnostic tests (RDT), positive result reflecting the presence of *Plasmodium falciparum* antigen in the blood. This prompted the initiation of an antimalarial treatment based on quinine injection at a rate of 25 mg/kg/day, which was discontinued after 3 days. Treatment was resumed 4 days later after the reappearance of symptoms. On the third day after resumption of treatment, corresponding to 10 days after the onset of symptoms, the patient was referred to Zinder National Hospital following a clinical aggravation marked by impaired consciousness. On arrival, clinical examination revealed a fever at 39°C, impaired consciousness with a Glasgow score of 11, mucous membrane pallor, conjunctival jaundice (Figure 1(a)), tachypnea with 40 cycles/min, bronchial rales, and tachycardia at 120 beats/min.

**Figure 2:**
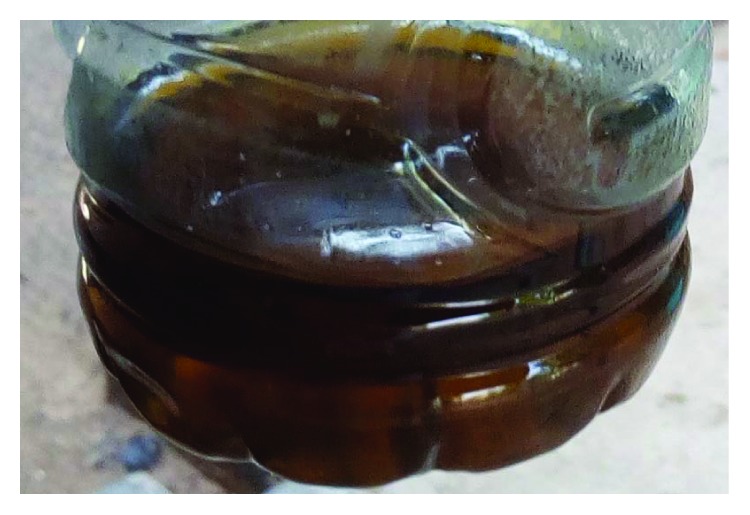
Coca-Cola colored urine.

The haemodynamic state was stable with a blood pressure of 120/80 mm Hg. Urine was Coca-Cola colored (Figure 2), which led to the diagnosis of BWF. Test strip urine examination revealed hemoglobinuria at 2 plus, bilirubinuria 3 plus, proteinuria 2 plus, and absence of urobilinogens, red blood cells, and leucocytes. The malaria RDT was positive, while the thick peripheral blood smear was negative. Biological examinations showed leucocytosis of 15000 elements/mm^3^, normochromic anaemia with a hemoglobin level of 4 g/dl, and a normal platelet count of 194,000/mm^3^. The direct Coombs test was negative. Urea: 74.9 mmol/L, serum creatinine: 559.6 *μ*mol/L, proteinuria: 0.56 g/24 h, ASAT: 334 IU/L, ALAT: 185 IU/L, CRP: 92 mg/L, blood ionogram: 128 mEq/L of sodium and 5 mEq/L of potassium, TP: 100%, INR: 0.99, TCA: 29.5, TQ: 12 sec, and a blood bilirubin level of 200 mg/L predominantly unconjugated. Abdominal ultrasound showed normal-sized kidneys, discrete renal dedifferentiation, and vesicular sludge. This presentation required dialysis for uremic syndrome. The patient received 3 hemodialysis sessions with transfusion of erythrocyte concentrates. The antimalarial treatment was continued with an intramuscular artemether derivative. Clinical status and renal function normalized after 7 days. The patient was discharged cured.

### 2.2. Second Observation

A 35-year-old woman with no particular history was hospitalized for a headache, fever, jaundice (Figure 1(b)), and impaired consciousness after 2 days of quinine injection administered at her home. On admission, clinical examination indicated a fever of 39.6°C, a weight of 60 kg, a Glasgow score of 10, a respiratory rate of 31 cycles/minute, a thick negative peripheral blood smear, but a malaria RDT positive for *P. falciparum*. The hemoglobin concentration was 5 g/dl, and the blood glucose level was 3.9 mmol/L. Urinary catheter insertion revealed port wine-colored urine, which prompted the BWF diagnosis. The urine test strip indicated hemoglobinuria at 2 plus; bilirubinuria 3 plus; proteinuria 2 plus; absence of urobilinogens, red blood cells, and leukocytes; creatinemia of 450 *μ*mol/L; and urea of 31.39 mmol; and quinine discontinuation was followed by treatment with intramuscularly injected artemether at a dose of 3.2 mg/kg daily on day 1 and 1.6 mg/kg daily from day 2 to day 5. The patient had three sessions of extrarenal purification and transfusion with 4,450 ml bags of blood. Evolution was favorable with clinical improvement and recovery of renal function after 5 days.

### 2.3. Third Observation

A 42-year-old man with no particular history was hospitalized for drowsiness, 38.9°C fever and a heart rate of 140 beats/minute. He weighed 55 kg. The thick peripheral blood smear was positive with 17,400 *Plasmodium falciparum* trophozoites per mm^3^. The hemoglobin concentration was 10.3 g/dl, and blood glucose was 9.4 mmol/L. Quinine treatment at a dose of 25 mg/kg per 24 hours was instituted in the emergency department. Obnubilation-type consciousness disorders and conjunctival jaundice appeared after 12 hours of hospitalization. Even though the patient was afebrile, the diagnosis of BWF was evoked due to the port wine color of his urine. The emergency bioassay showed a creatinemia of 720 *μ*mol, urea of 28 mmol, and hemoglobin of 5.5 g/dl. Quinine was immediately stopped, in favor of intramuscularly injected artemether prescribed at a dose of 3.2 mg/kg daily on day 1 and 1.6 mg/kg daily from day 2 to day 5. Unfortunately, the patient only received the first dose because he died less than 24 hours after admission of shock in coma and with anuria.

### 2.4. Fourth Observation

A 38-year-old man with no particular history was hospitalized for fever and jaundice. At admission, his temperature was 38.5°C with a Glasgow score of 11/15, a weight of 62 kg, a respiratory rate of 28 cycles/minute, and a heart rate of 100 beats/minute. The thick peripheral blood smear was negative, but the rapid diagnostic test was positive for *P. falciparum.* Hemoglobin concentration was 9.0 g/dl, blood glucose was 5.6 mmol/L, and serum creatinemia was 1500 *μ*mol/L. The patient had received a first dose of quinine at a dose of 25 mg/kg/24 hours the previous day. An oligoanuria with port wine-colored urine was noted. Quinine was discontinued and replaced with intramuscularly injected artemether at a dose of 3.2 mg/kg daily on day 1 and 1.6 mg/kg daily from day 2 to day 5. The patient had 4 hemodialysis sessions. The evolution was favorable with progressive improvement of renal function.

## 3. Discussion

BWF is found in areas where malaria is endemic [1, 11]. Several authors have reported on its recrudescence, especially in Africa [9, 12]. Of a total of 3170 cases of malaria, Olupot-Olupot et al. [12] had collected 394 cases of BWF (12.4%). The four patients in this study were admitted in the month of September alone, despite the disease having become rare in our hospital. The average age in our sample is 39 years old. No distinction by sex or age was noted in the occurrence of BWF [11]. The predominance of *P. falciparum* was also reported by many authors [9]. However, in 1988, Katongole-Mbidde et al. [13] published a case of blackwater fever due to *Plasmodium vivax*. In 2016, Baber et al. [2] described the first case of BWF due to *Plasmodium knowlesi*, and Madhuri et al. [14] had described a case of BWF due to *Plasmodium malariae*.

Treatment with quinine was the triggering factor in our patients as is noted by most authors [4, 12–15]. However, in 2014, Lon et al. described the first case of artemisinin-treated BWF in simple malaria [15], and more recently, other authors have reported cases of BWF following treatment with artemisinin derivatives [2, 7]. In the AQUAMAT trial, comparing the therapeutic efficacy of artesunate versus quinine in the treatment of severe malaria in African children, artesunate was associated with the occurrence of BWF in 18/2597 (0.7%) and quinine in 30/2591 (1.2%) [16]. In eastern Uganda, Olupot-Olupot et al. [12] thought that the increase of BWF could be related to the introduction of artemisinin-based combination therapies.

BWF pathophysiology is complex and not yet well known, but according to some authors, the disease is secondary to double red blood cell sensitivity to antimalarial drugs and *Plasmodium falciparum*, causing acute hemolysis [1, 2]. Intravascular hemolysis occurs after taking the offending drug [4]. The released hemoglobin causes nephrotic obstruction, which leads to acute renal failure. Anaemia and degraded hemoglobin products contribute to coma development, or even patient death, in the absence of treatment [6]. The existence of a G6PD enzymatic deficiency is a determining factor in the occurrence of this hemolysis [4]. This analysis could not be performed in our patients. Nevertheless, there was no thrombocytopenia, and the negative direct Coombs test allowed us to eliminate autoimmune hemolysis or uremic hemolytic syndrome.

Clinically, the syndrome is well characterized [4]. Presentation is typically acute hepatonephritis occurring 24 to 48 hours after the administration of antimalarial drugs. Anaemia is severe from the start and intravascular hemolysis, occurring in a context of severe malaria, leading to oliguria, dark urine, abdominal pain, jaundice, hepatic splenomegaly, vomiting, and renal failure [4]. Renal involvement is thought to be secondary to acute tubular necrosis [4]. The port wine coloration of urine is the most consistent sign; Bodi et al. [17] in the Democratic Republic of the Congo (DRC) found this aspect in all patients. Other symptoms may be observed such as disturbances of consciousness, dyspnea, tachycardia, general malaise, and vertigo. Rapid onset and deterioration were described with immediate collapse, coma, and anuria [11]. Our patient died under these conditions. The time to onset of symptoms may be longer; in 2015, Thiongane et al. [3] described a case of BWF appearing 3 days after taking quinine. In our first observation, this delay was 10 days, associated, however, with the misuse of quinine because the duration of initial treatment was not respected. Quinine misuse is reported by many authors as a risk factor for BWF [16].

The very high lethality observed initially has today significantly decreased in hospitals [5, 17]. Current lethality figures reported by most authors vary from 23 to 26% [3]. In our patient, death occurred less than 24 hours after the onset of hemolysis as a result of shock, coma, and anuria. This swift form is well described in the literature [17].

BWF treatment is based on the discontinuation of quinine or other drugs that may trigger hemolysis, the use of artemisinin derivatives when they are not incriminated, blood transfusion, intravenous fluids, electrolytic re-equilibration, and, frequently, extrarenal purification [1–3, 11, 18, 19, 20].

Limitations of this study are technical. Indeed some explorations are not available in our context, including the search for G6PD deficiency and drug concentration dosages in the blood or urine.

## 4. Conclusion

BWF is a serious and rare complication of malaria and its treatment. It should be remembered in a patient undergoing malaria treatment admitted with jaundice, abdominal pain, and acute renal failure with port-colored urine. The disease seems to be more frequent in periods of high endemicity (rainy season in the Sahel). Treatment often requires extra-renal cleansing which is essential. Quinine incriminated in our patients is not the only drug involved in the occurrence of BWF. Several other drugs (amino alcohols, artemisinin derivatives, and lumefantrine) used as an alternative to this are also reported in the literature.
